# Supplementary material for: Electronic components embedded in a single graphene nanoribbon
Source: Nat Commun. 2017 Jul 25;8:119. doi: 10.1038/s41467-017-00195-2 (PMC5527019; doi:10.1038/s41467-017-00195-2)
Supplement: Supplementary file 1 — Supplementary Information_JWF ready [file 41467_2017_195_MOESM1_ESM.pdf]

File Name: Supplementary Information
Description: Supplementary Figures, Supplementary Notes, Supplementary Methods, Supplementary Discussion and Supplementary References.

File Name: Peer Review File
Description:

Supplementary Discussion

In order to investigate the effect of the five-membered ring, we have performed a tight-binding calculation on a type I junction and type II junction, both of a 5/7-GNR(6,6). The results are shown in Supplementary Fig. 1. Note that these calculations do not take electron-electron interactions into account, so they do not return the characteristic spin-splitting of the edge states that we find in DFT. Also, overlap has been switched off here in order to assess the symmetry of the energy spectrum.

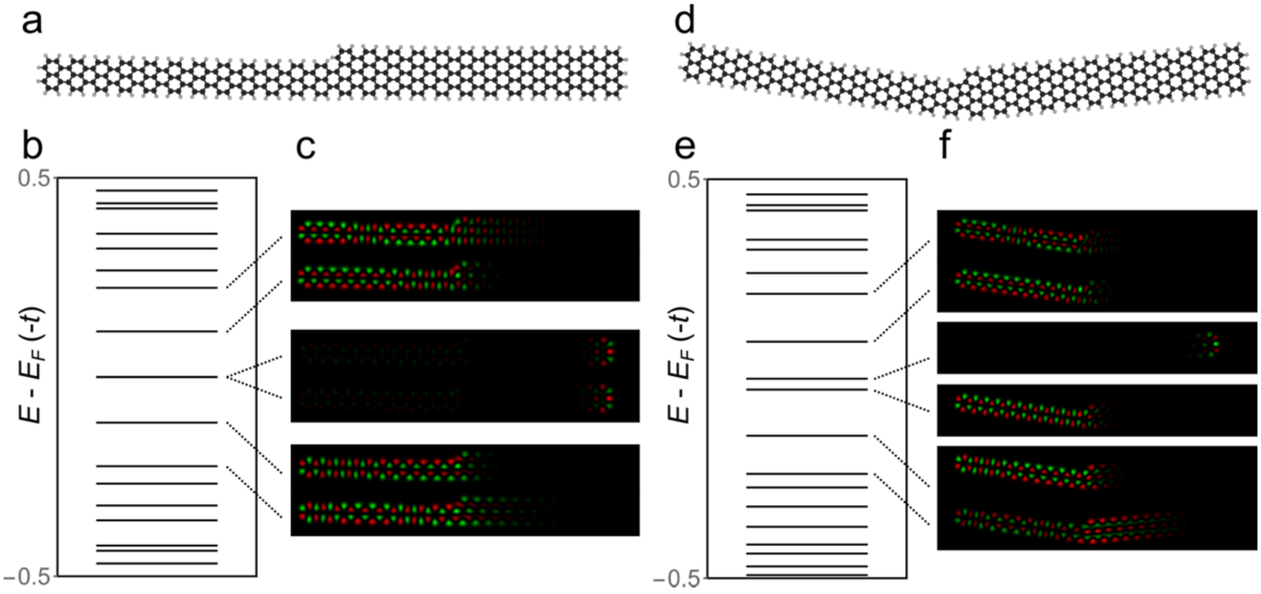

Supplementary Figure 1 | Calculated energy levels and molecular orbitals for the 5/7-GNR(6,6). a, Model of the junction of type I. **b,** Calculated energy level spectrum for the junction of type I. **c**, Calculated frontier orbitals for the junction of type I. **d**, Model of the junction of type II. **e,** Calculated energy level spectrum for the junction of type II. **f**, Calculated frontier orbitals for the junction of type II.

As can be seen in the cove-edge case (geometry in Supplementary Fig. 1a), the spectrum is completely symmetric around the Fermi energy (Supplementary Fig. 1b). The two end-localized states are degenerate at exactly the Fermi level. The orbitals corresponding to the end-localized states show a strongly localized feature on the 7-GNR end and a feature delocalized over the 5-GNR segment (Supplementary Fig. 1c). The frontier orbitals show a predominant localization on the 5-GNR segments in the same way as obtained from DFT calculations discussed in the manuscript.

The MO diagram of the 6,6 ribbon with type II junction (Supplementary Fig. 1d), shows a significant asymmetry (Supplementary Fig. 1e). The end state localized on the 7-GNR end remains unperturbed at the Fermi energy, whereas the other end state now resides at lower energy due to the extended delocalization (Supplementary Fig. 1f). Apparently, the extra bond improves the electronic coupling of the 7-GNR end state with the 5-GNR bulk states and the corresponding breaking of the bipartite symmetry of the lattice allows the newly formed end state to lower its energy.

We conclude that the result from DFT that the energy of the "connecting" end state is lowered upon contacting a 5-GNR segment can in fact be cast into the framework of breaking of the bipartite lattice through introduction of the five-membered ring.

We now switch the discussion to the observed current decay behavior in the lifting experiments. We note that in the calculations, the current decay parameter is often underestimated. We believe this to be due to the absence of explicit electron-electron interactions in the tight binding model, which results in an underestimation of the band gap. In the case of non-resonant transport, this error translates into an underestimation of β, since the energetic distance between the Fermi level of the tip and the HOMO and LUMO is reduced. An additional effect is that the end-localized state may contribute to the transport, but due to its confined nature, its contribution may quickly cancel upon lifting. We believe this to be the reason why in the calculation of the conductance in Figure 4a, β is higher at the beginning of the $I(z)$ curve, where the end state still plays a role. After lifting to Δz > 1 nm, the current decay converges to a monoexponential decay, the β value of which underestimates the real value due to the underestimated band gap. The fact that the same end state feature cannot be seen in the experiments could be due to the ribbon already being slightly lifted from the substrate upon being contacted, meaning that the zero height is shifted with respect to the zero in the calculations. Unfortunately, we cannot measure the absolute value of the tip height in STM.

We have tried different levels for the Fermi energy and found that a value of 50meV below the HOMO peak was optimal for reproducing the $\ln(I/\mathrm{nA})(z)$ experiments. This is equivalent to the junctions having a charge that is slightly larger than one unit charge, in agreement with the conclusions from DFT calculations (see Supplementary Note 2).

Supplementary Note 1: Additional spectroscopy results on ribbons containing a single junction

In addition to 5/7-GNR(2,2), we have characterized the 5/7-GNR(4,4) that is exactly twice the length and also has a single-junction. The spectroscopy results are shown in comparison with the results from the 5/7-GNR(2,2) in Supplementary Fig. 2.

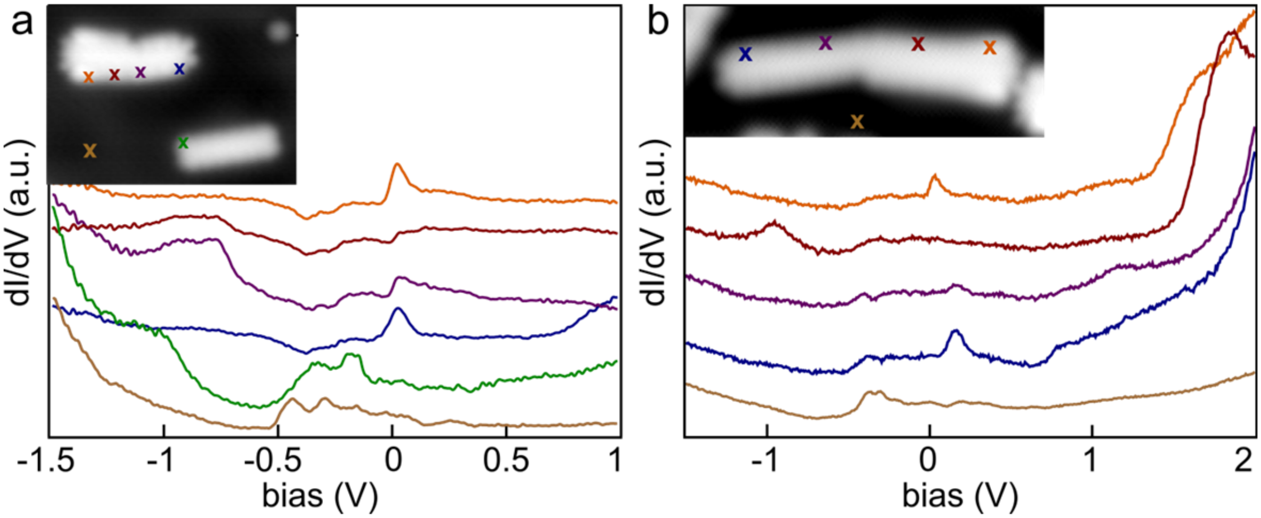

Supplementary Figure 2 | Differential conductance spectra on two single-junction nanoribbons. a, Differential conductance spectra of a 5/7-GNR(2,2), as well as a spectrum recorded on 5-GNR(3). These are the same GNRs as shown in Figure 2a. **b,** Differential conductance spectra of a 5/7-GNR(4,4). The insets show constant-current STM topographs *V* = 0.1 V and *I* = 200 pA.

The same general features as in the shorter analogue are observed: a low-bias state localized at the free end and the narrow segment, as well as a negative bias resonance (-0.9 V) localized near the interface region. Additionally, as spectra were obtained for a larger bias region, the onset of unoccupied states could be resolved. Specifically, for the wide segment, no resonance is observed up to 1.6 V, whereas the narrow end already displays an onset at around 0.9 V.

Supplementary Note 2: DFT results on experimentally characterized junctions

For all ribbons, calculations were performed for different charge states, to see how the local density of states and simulated differential conductance maps are affected.

The density of states (DOS) shown below were calculated in the following way. The eigenenergies of both spin channels as given by the DFT calculations were broadened with Lorentzians with FWHM of 0.1 eV. The sum of these Lorentzians represents the density of states.

The simulated differential conductance maps were generated by the following approach: For all eigenstates within 2 eV of the Fermi level, the value of the wave function is determined at a height of 3.5 Å above the molecular plane. The wave functions are squared to obtain the orbital density. The orbital images are subsequently summed with weights proportional to the broadened Lorentzian at that energy to obtain the simulated differential conductance map.

In general, we find experimentally that any low-bias states originating from the 7-GNR ends are always at slightly positive bias (approximately 50 mV). This result is consistently obtained in the DFT calculations on heterostructure with charge q = +1, and not in the results for charge states q = 0 and q = +2. For this reason, we believe all heterostructures to have a charge of q = +1. This is also consistent with known doping of bulk graphene on Au(111) substrate.

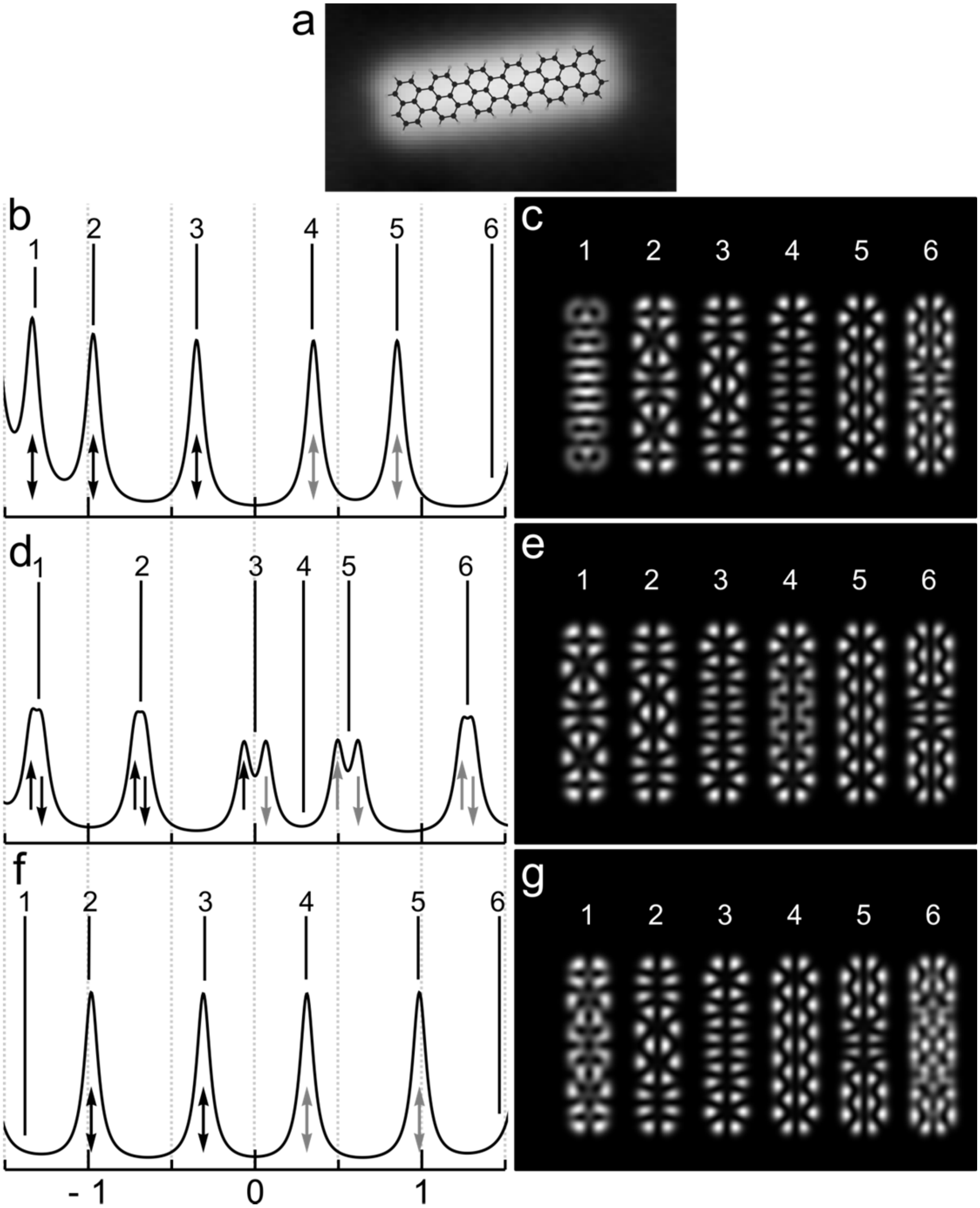

Supplementary Figure 3 | Calculated spectra and simulated differential conductance maps of the 5-GNR(3). a, Model of the junction. **b,d,f**, Calculated DOS for q = +2, +1 and 0, respectively. **c,e,g**, Simulated differential conductance maps at the energies indicated in b,d,f respectively.

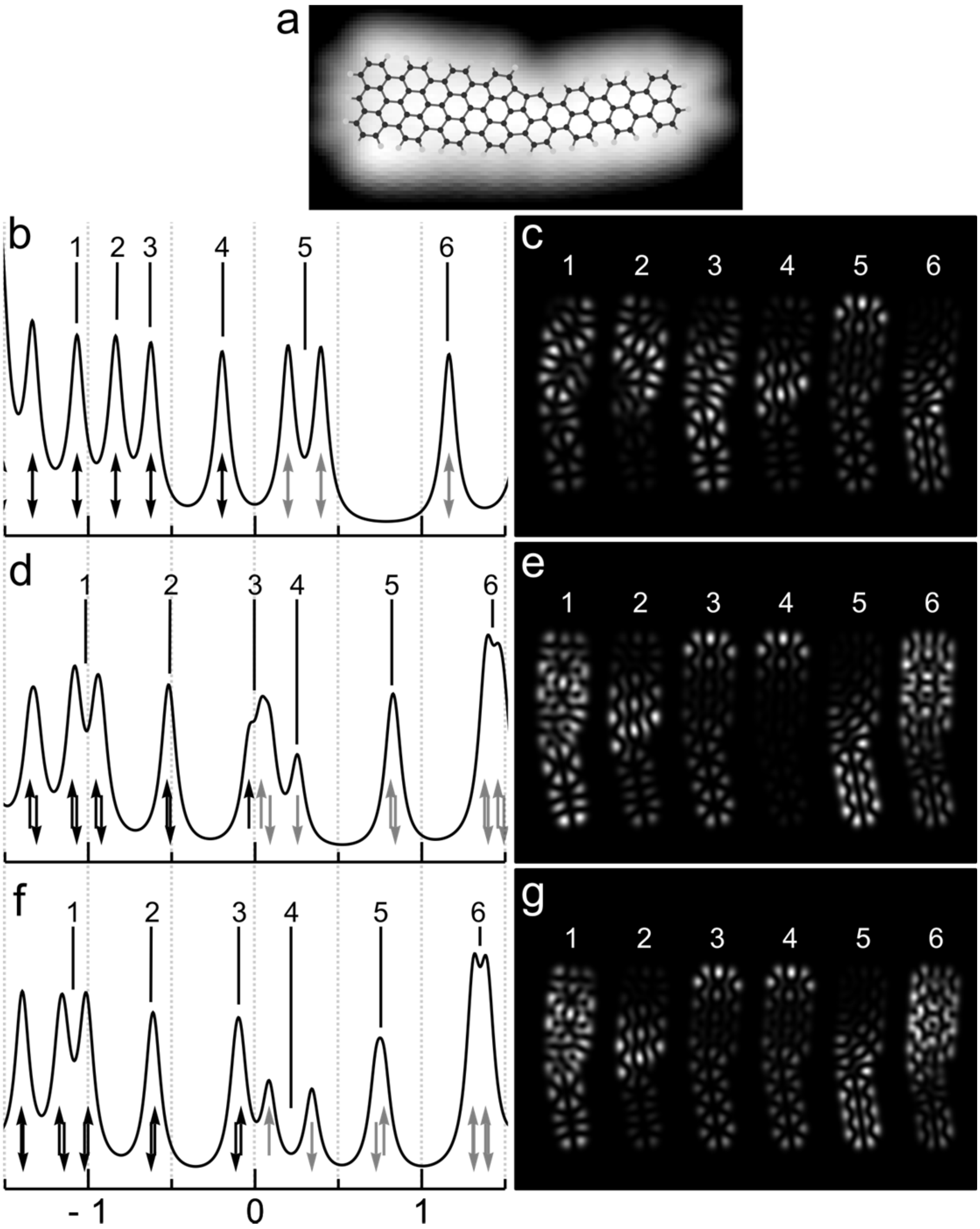

Supplementary Figure 4 | Calculated spectra and simulated differential conductance maps of the 5/7-GNR(2,2) junction. a, Model of the junction. **b,d,f**, Calculated DOS for q = +2, +1 and 0, respectively. **c,e,g**, Simulated differential conductance maps at the energies indicated in b,d,f respectively.

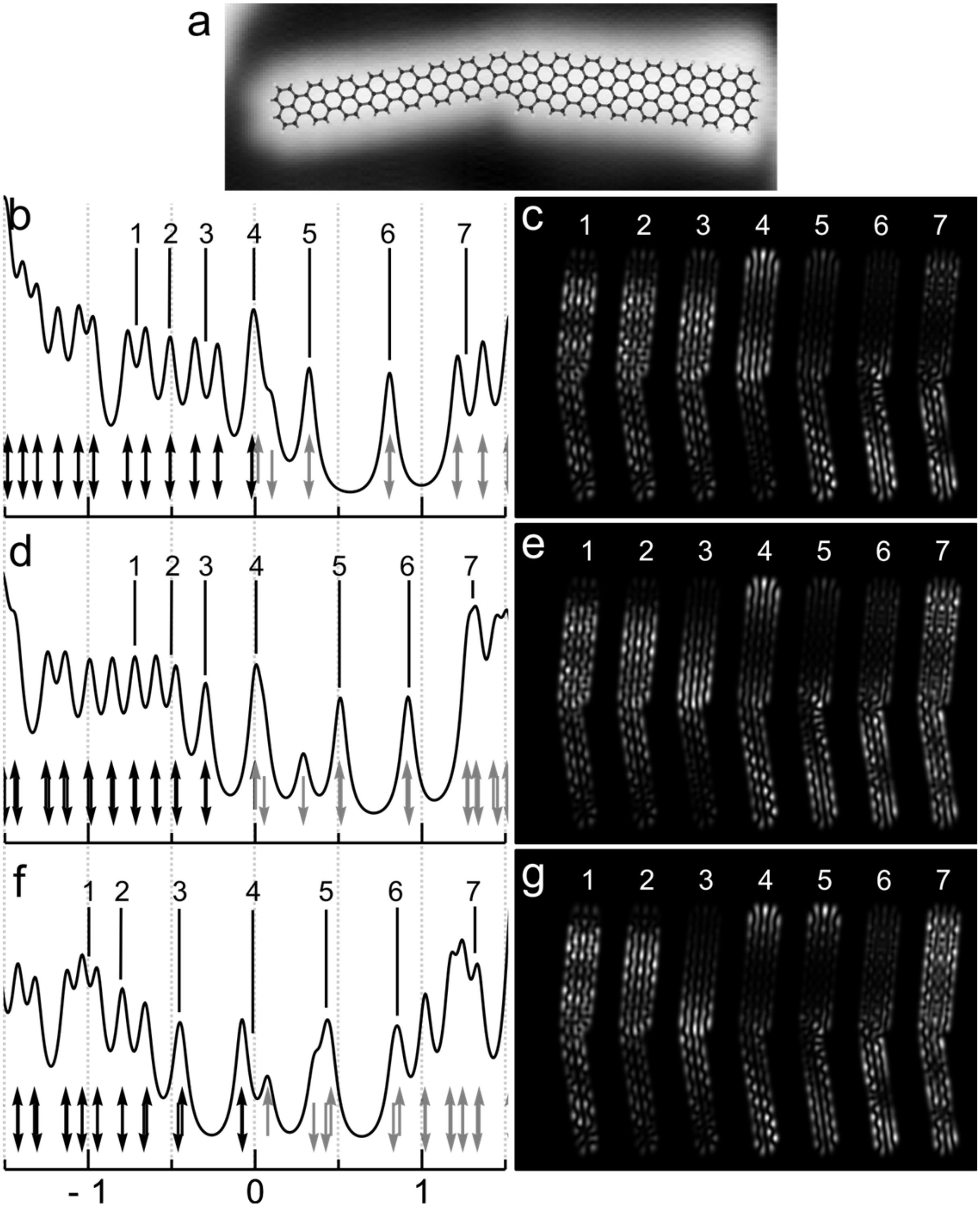

Supplementary Figure 5 | Calculated spectra and simulated differential conductance maps of the 5/7-GNR(4,4) junction. a, Model of the junction. **b,d,f**, Calculated DOS for q = +2, +1 and 0, respectively. **c,e,g**, Simulated differential conductance maps at the energies indicated in b,d,f respectively.

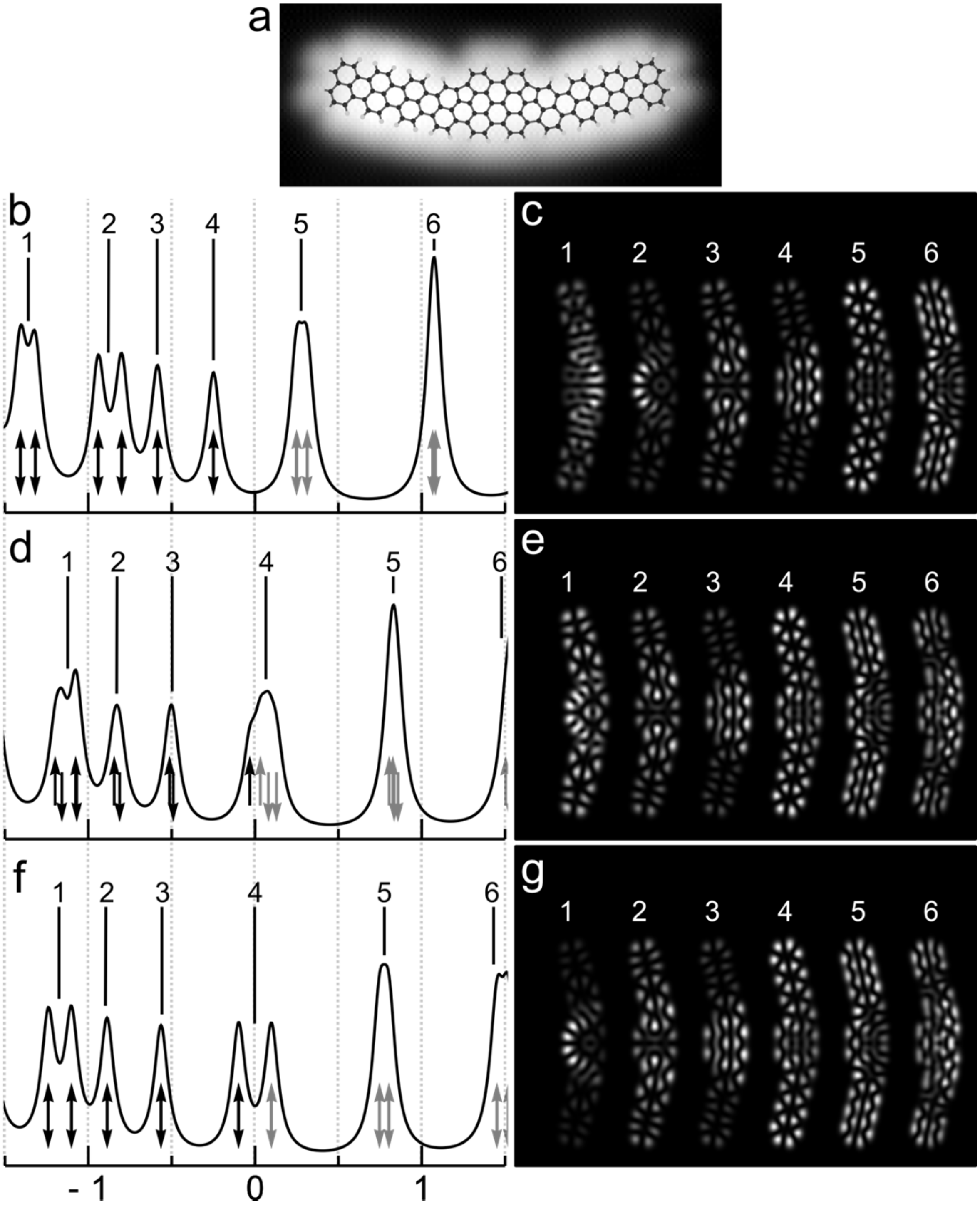

Supplementary Figure 6 | Calculated spectra and simulated differential conductance maps of the 5/7/5-GNR(2,1,2) junction. **a**, Model of the junction. **b,d,f**, Calculated DOS for q = +2, +1 and 0, respectively. **c,e,g**, Simulated differential conductance maps at the energies indicated in b,d,f respectively.

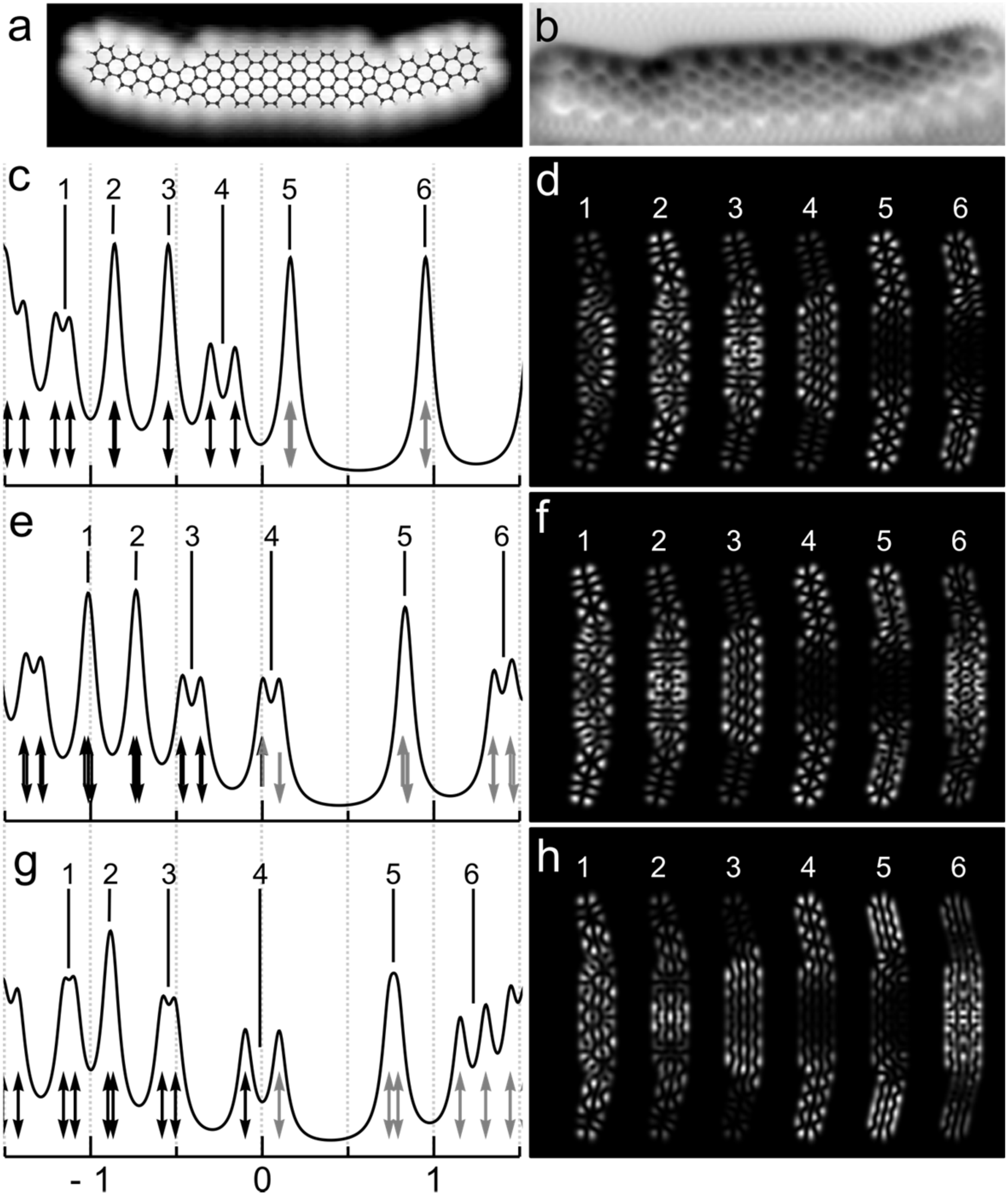

Supplementary Figure 7 | Calculated spectra and simulated differential conductance maps of the 5/7/5-GNR(2,3,2) junction. **a**, Model of the junction. **b,** corresponding constant height AFM image. **c,e,g**, Calculated DOS for q = +2, +1 and 0, respectively. **d,f,h**, Simulated differential conductance maps at the energies indicated in b,d,f respectively.

Supplementary Note 3: Additional lifting experiments

Two types of conductance experiments were performed, *I*(*z*) measurements and *I*(*V*) spectroscopy experiments. The first step in these experiments is to pick up a GNR with the STM tip, which is achieved by lowering the tip over one of the termini until the nanoribbon jumps to contact (as described in the methods section). *I*(*z*) lifting experiments were conducted with a voltage bias of 10 mV. A successful *I*(*z*) experiment consists of a number of cycles of tip retraction and approach to the surface. *I*(*z*) curves for all cycles and for both retraction and approach are superimposed. For most ribbons, lifting experiments were conducted at both termini. We note that pick-up is not always successful, with the success rate depending strongly on the condition of the tip and differing for the side of the ribbon which was contacted. Overall we found a significantly higher success rate of contacting the 7-GNR sides. Therefore, we did not always succeed in measuring *I*(*z*) for both termini. Furthermore, in a few instances, ribbons were lifted too far off the surface, resulting in the ribbon fully detaching from the surface and attaching to the tip. This was evidenced by a sudden drop in current, often accompanied by a change in contrast during subsequent scans, and failure to relocate the nanoribbon on the surface. We also encountered many "drop-downs", sometimes during lifting or spectroscopy experiments, evidenced by a similar drop in current but relocation of the nanoribbon in subsequent scans. In these cases, the ribbons were found laterally displaced in the direction of the terminus which was picked up, in agreement with Koch *et al*.[1]

For all curves, the corresponding ln(*I*/nA)(*z*) were calculated. In the following figures, we have displayed the *I*(*z*) curves in red and green, with the red curves corresponding to the segment with the most 7-GNR "character" (7-GNR side or shortest 5-GNR side) and the green curves corresponding to segments with the most 5-GNR "character" (5-GNR side or shortest 7-GNR side). ln(*I*/nA)(*z*) curves are displayed in gray, or in dark red and dark green, corresponding to the same respective termini. The GNRs are shown in insets, with the tip position used for lifting experiments indicated by a red or green cross, respectively.

For most experimental ln(*I*/nA)(*z*) curves we calculated the parameter $\beta = \frac{\partial}{\partial z}\ln I$ by fitting to the linear regimes of the decay. The resulting values are shown immediately adjacent to the ln(*I*/nA)(z) curves. For all GNRs, we calculated the *I*(*z*) and ln(*I*/nA)(*z*) curves by means of the non-equilibrium Green's function method (NEGF), applied to a tight-binding representation of the nanoribbon. The results from these calculations are shown next to the experimental results. Again, we have used green/red for lifting from the two termini, and dark green/dark red for the ln(*I*/nA)(*z*) curves. An integral part of these calculations is the description of the coupling of the nanoribbon to the surface, which requires an estimate of the geometry of the nanoribbon being lifted off the surface while being attached to the tip. Therefore, we have modeled the geometry of the nanoribbons by minimizing the mechanical energy for each tip height (details are given in the Supplementary Methods). We have interspersed simulated geometries of the ribbons, to give an impression of how the nanoribbons are "peeled off" from the surface in the lifting process, and to show what geometries were used to obtain the simulated transport curve results. Details on tight binding and transport calculations are given in the Supplementary Methods.

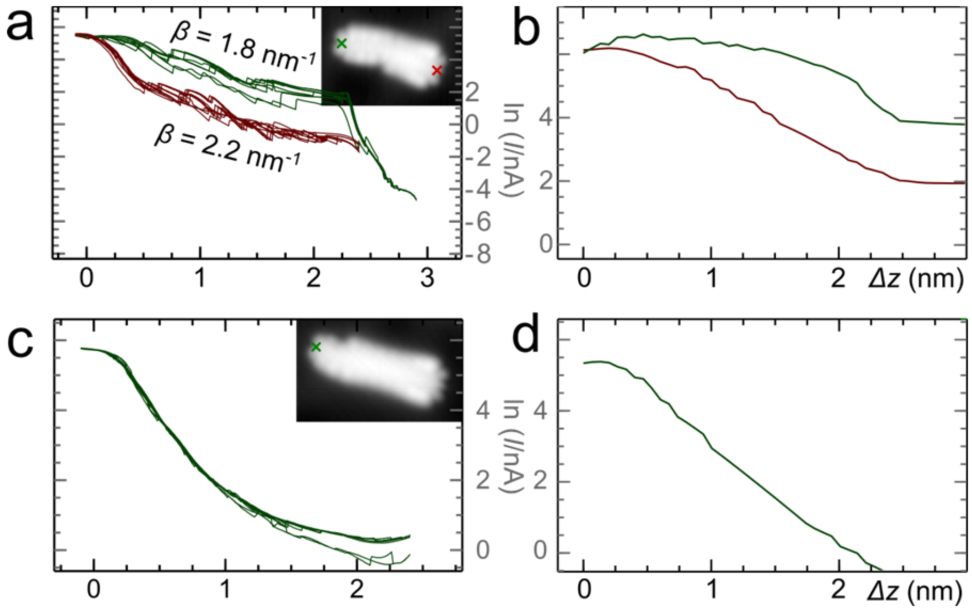

Supplementary Figure 8 | Lifting experiments on single junction GNRs of total length *n* = 4 monomers. **a,** experimental and **b**, simulated *I*(*z*) and ln(*I*/nA)(*z*) for the 5/7-GNR(2,2) **c**, experimental and **d**, simulated *I*(*z*) and ln(*I*/nA)(*z*) for the 5/7-GNR(1,3).

Supplementary Fig. 8 shows the results of lifting experiment on junctions in which the total length is 4 monomers. Lifting from the 7-GNR side in the 5/7-GNR(2,2) displays a notable transition from fast decay (Δz < 0.4 nm) to slow decay (Δz > 0.4 nm). For Δz > 2.3 nm (≈ 70 % of the nanoribbon length) the ln(*I*/nA)(*z*) drops sharply for the 5-GNR side, which we found to be indicative for near-detachment.

Supplementary Fig. 9 shows the results of lifting experiment on single junctions in which the total length is 5 monomers. For this length, we have performed experiments on both the 5/7-GNR(2,3) and 5/7-GNR(3,2). The junctions may be considered chiral when constrained to a two-dimensional surface, and we have performed experiments on both enantiomers for both junctions. Lifting geometries for both ribbons when picked up from either side are shown in Supplementary Fig. 9b and d. As seen in Supplementary Fig. 9a and c, lifting from the 5-GNR end results in an initial hump after which the ln(*I*/nA)(*z*) curves down. Lifting experiments from the 7-GNR terminus show a very sharp transition from fast decay to slow decay in the logarithm of the current. For the 5/7-GNR(3,2), this transition occurs after 0.7 nm, whereas for the 5/7-GNR(2,3) the transition is slightly later, at 1.0 nm. This is evidently a result of the increased length of the 5-GNR segment. A similar offset in transition is obtained in the simulations, as can be seen in Supplementary Fig. 9b and d. Supplementary Fig. 9e shows an atomically resolved AFM image, which was recorded with a nanoribbon-passivated tip (achieved through pick-up of a nanoribbon). Supplementary Fig. 10 shows more *I*(*z*) curves obtained on longer ribbons.

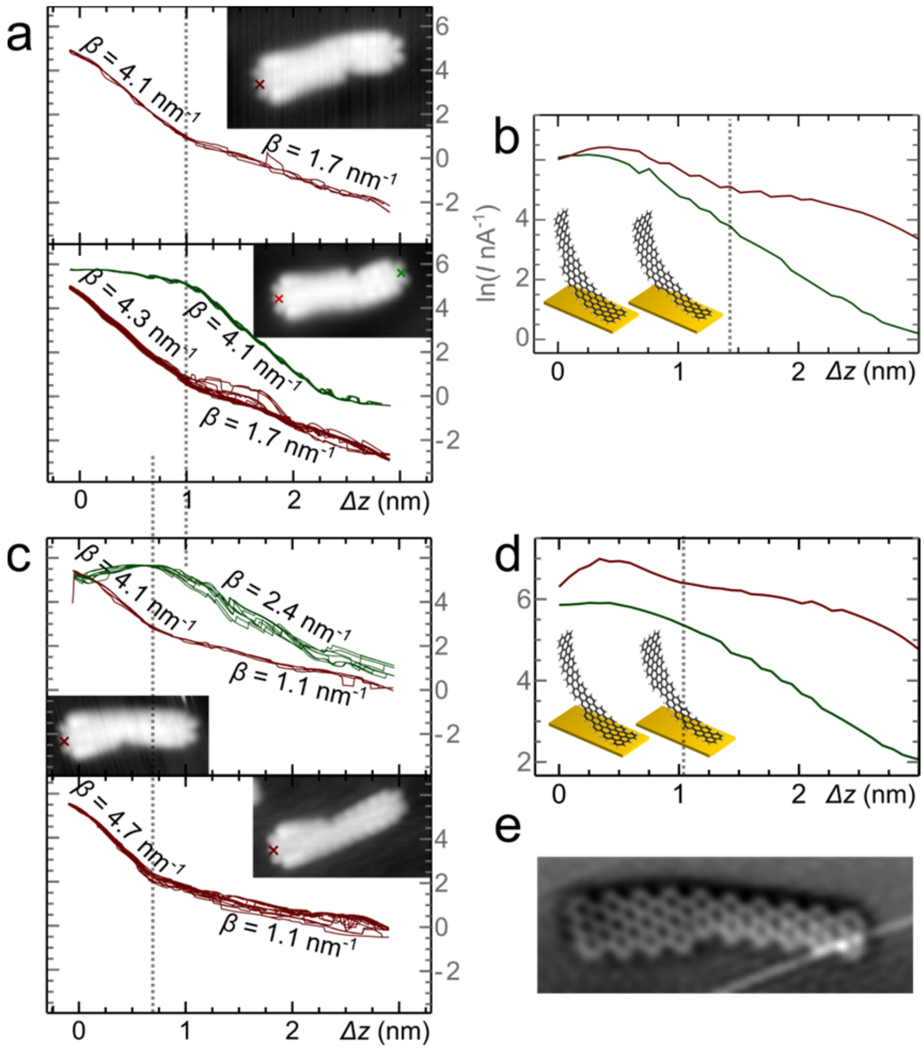

Supplementary Figure 9 | Lifting experiments on single junction GNR of total length *n* = 5 monomers. **a**, experimental and **b**, simulated ln(*I*/nA)(*z*) for the 5/7-GNR(2,3). The inset in **b** shows simulated lifting geometries of the 5/7-GNR(2,3). **c**, experimental and **d**, simulated ln(*I*/nA)(*z*) forthe 5/7-GNR(3,2). The inset in **d** shows simulated lifting geometries of the 5/7-GNR(3,2). **e** AFM image of the 5/7-GNR(3,2), *z*-offset for AFM image - 40 pm w.r.t a STM set-point of *V* = 100 mV, *I* = 20 pA.

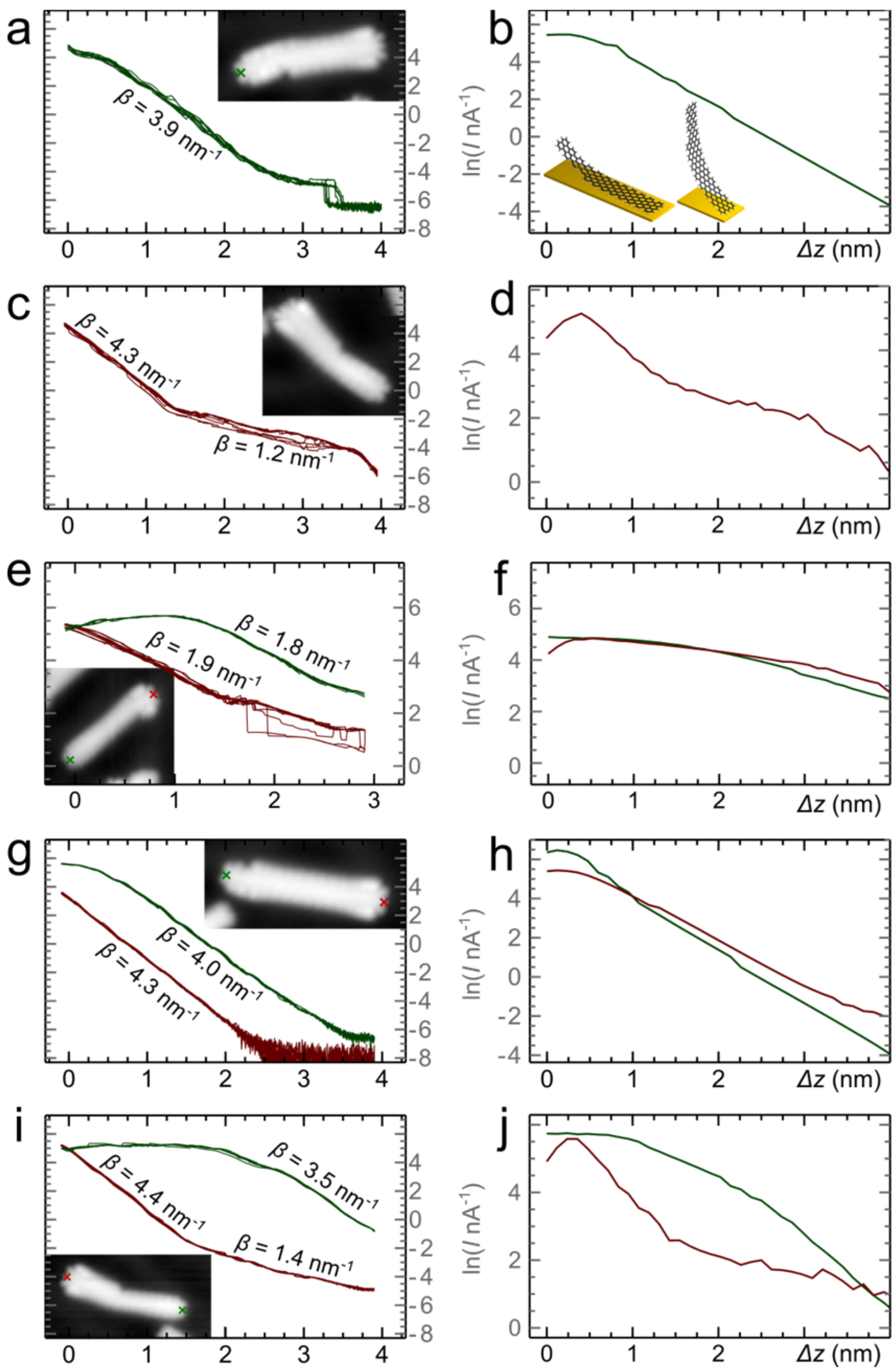

Supplementary Figure 10 | Lifting experiments on single junction GNR of total length *n* > 5 monomers. **a,** experimental and **b,** simulated *I*(*z*) and ln(*I*/nA)(z) for the 5/7-GNR(2,4). The inset shows simulated lifting geometries of the 5/7-GNR(2,4). **c**, experimental and **d**, simulated ln(*I*/nA)(*z*) for the 5/7-GNR(3,3). **e**, experimental and **f**, simulated ln(*I*/nA)(*z*) for the 5/7-GNR(5,1). **g**, experimental and **h**, simulated ln(*I*/nA)(*z*) for the 5/7-GNR(1,5). **i**, experimental and **j**, simulated ln(*I*/nA)(*z*) for the 5/7-GNR(4,3).

Supplementary Methods

Transport calculations were performed using the non-equilibrium Green's function method. Since the contact sizes on the substrate prohibit using DFT implementations of the NEGF formalism, we modeled the electronic structure of the nanoribbon with the cheaper tight-binding model instead. Tight binding has been shown to be particularly well applicable on large, conjugated systems like graphene nanoribbons.[2] We use a nearest-neighbour tight binding model defined by the Hamiltonian given in equation 1

(1) $$\hat{H} = \varepsilon_0 \sum_{i}^{atoms} a_i^\dagger a_i - t \sum_{i,j}^{pairs} (a_i^\dagger a_j + a_j^\dagger a_i)\, \delta_{i,j\in nn}$$

where we set the on-site energy ε_0 = -5.0 eV, nearest neighbor hopping integral t = 2.8 eV and nearest-neighbor overlap s = 0.15.[3] The transport through the system, i.e. the leads and the junction, is calculated based on the Non-Equilibrium Green's Function (NEGF) formalism. Using this formalism, the calculation of the transport device plus two semi-infinite electrodes is reduced to a calculation performed within the Hilbert space of the transport region only. The central quantity in the calculation is the retarded Green's function, which is an operator within that Hilbert space, given as

(2) $$G^+(\varepsilon) = \frac{1}{\varepsilon S - H - \Sigma^L(\varepsilon) - \Sigma^R(\varepsilon)}$$

Here, $\Sigma^{L,R}$ are the self-energies of the left and right lead – these have nonzero elements only on the sites which couple to the left and right electrodes respectively. These self-energies can be divided into a real and an imaginary part

(3) $$\Sigma^{L,R}(\varepsilon) = \Lambda(\varepsilon)^{L,R} - \frac{i}{2}\Gamma(\varepsilon)^{L,R}$$

which are in principle energy dependent. However, for gold leads, the coupling between the leads and the states in the molecule varies only slowly with energy. This leads to energy-independent self-energies, which is known as the wide-band limit (WBL).

Having defined the coupling to the leads, the transmission is given by equation 4

(4) $$T(\varepsilon) = Tr\{\Gamma^L G^+ \Gamma^R G^-\}$$

where G^- is the advanced Green's function, which is the Hermitian conjugate of G^+. Applying the Landauer formula,[4] the current in the low-temperature limit is given by

(5) $$I = \frac{2e}{h}\int_{\mu_L}^{\mu_R} T(\varepsilon)d\varepsilon$$

with $\mu_{L,R}$ the chemical potentials of the leads.

Modeling of the leads in the transport calculations

The leads in the lifting experiments are the STM tip and the gold surface. The STM tip is expected to couple to a single atom site, with a capacitance much smaller than that of the gold surface. It can therefore be modeled, taking it to be the left contact, and coupling to atom site *i* as

$$\Sigma_{ii}^{L} = -\frac{i}{2}\Gamma_{ii}^{STM} \tag{6}$$

The coupling of the molecule to the gold substrate is expected to be exponentially decaying with the distance between an atom and the substrate, typically with a distance of about the radius of a gold atom, which is 1.5 Å. At the gold-ribbon interface, various physical processes are responsible for a relative shift of the energies in both.[5] This shift is captured in the real part Λ of the self-energy. The self-energy of the gold substrate is therefore modeled by

$$\Sigma_{ii}^{R} = \left(\Lambda_{ii}^{Substrate} - \frac{i}{2}\Gamma_{ii}^{Substrate}\right)e^{-\frac{z_i}{z_0}} \tag{7}$$

with z_i the height with respect to the ribbon lying flat on the substrate of atom *i.* Other parameters needed for the transport calculations are the capacitive coupling between the gold substrate and the graphene ribbon. The Fermi energy of the Au(111) is about -5.3 eV [6], and that of graphene (*in vacuo*) about -4.5 eV.[7,8] Given the fact that the Fermi energy of graphene on gold lies about 0.4 eV above that of gold,[9] we conclude that it lies 0.4 eV below that of graphene *in vacuo.* We therefore take Λ to be 0.4 eV. The broadening for both leads is set to 100 meV.[10] Lastly, an additional broadening factor of the same value is applied to each site to account for broadening due to various mechanisms.[11]

Modeling the lifting experiments

For the shape of the ribbon we have used a simple parametrized form which combines a straight stretch (possibly of zero length) close to the tip with a circular segment close to the substrate, and minimized the mechanical energy with respect to the radius of curvature, using a bending energy of 3.9 eV $Å^{-2}$ per atom.[12] Finally, a binding energy of 40 meV per atom[13] was used, together with an energy penalty linear with the sliding distance when lifting the tip of 0.1 eV $Å^{-1}$.[14]

Supplementary References

1. Koch, M., Ample, F., Joachim, C. & Grill, L. Voltage-dependent conductance of a single graphene nanoribbon. *Nat. Nanotechnol.* **7,** 713–717 (2012).

2. Wakabayashi, K., Takane, Y., Yamamoto, M. & Sigrist, M. Electronic transport properties of graphene nanoribbons. *New J. Phys.* **11,** 95016 (2009).

3. Kundu, R. Tight-Binding Parameters for Graphene. *Mod. Phys. Lett. B* **25,** 163–173 (2011).

4. Haug, H. & Jauho, A. P. *Quantum Kinetics in Transport and Optics of Semiconductors*. (Springer Berlin Heidelberg, 2007).

5. Ishii, H. *et al.* Energy level alignment and band bending at model interfaces of organic electroluminescent devices. *J. Lumin.* **87–89,** 61–65 (2000).

6. Hüfner, S. *Photoelectron Spectroscopy: Principles and Applications*. (Springer Berlin, 2003).

7. Christodoulou, C. *et al.* Tuning the Work Function of Graphene-on-Quartz with a High Weight Molecular Acceptor. *J. Phys. Chem. C* **118,** 4784–4790 (2014).

8. Barone, V., Peralta, J. E., Uddin, J. & Scuseria, G. E. Screened exchange hybrid density-functional study of the work function of pristine and doped single-walled carbon nanotubes. *J. Chem. Phys.* **124,** 24709 (2006).

9. Sławiniska, J., Wlasny, I., Dabrowski, P., Klusek, Z. & Zasada, I. Doping domains in graphene on gold substrates: First-principles and scanning tunneling spectroscopy studies. *Phys. Rev. B* **85,** 235430 (2012).

10. Hoggard, A. *et al.* Using the Plasmon Linewidth To Calculate the Time and Efficiency of Electron Transfer between Gold Nanorods and Graphene. *ACS Nano* **7,** 11209–11217 (2013).

11. Funk, H., Knorr, A., Wendler, F. & Malic, E. Microscopic view on Landau level broadening mechanisms in graphene. *Phys. Rev. B* **92,** 205428 (2015).

12. Kudin, K. N., Scuseria, G. E. & Yakobson, B. I. C2F, BN, and C nanoshell elasticity from ab initio computations. *Phys. Rev. B* **64,** 235406 (2001).

13. Giovannetti, G. *et al.* Doping Graphene with Metal Contacts. *Phys. Rev. Lett.* **101,** 26803 (2008).

14. Kawai, S. *et al.* Superlubricity of graphene nanoribbons on gold surfaces. *Science.* **351,** 957–961 (2016).
